# Thiamine Supplementation Improves Survival and Body Condition of Hatchery-Reared Steelhead (*Oncorhynchus mykiss)* in Oregon

**DOI:** 10.3390/vetsci10020156

**Published:** 2023-02-14

**Authors:** Aimee N. Reed, Freya E. Rowland, Jennifer A. Krajcik, Donald E. Tillitt

**Affiliations:** 1 Oregon Department of Fish and Wildlife, Fish Health Services, OSU 226 Nash Hall, Corvallis, OR 97331, USA; 2 U.S. Geological Survey, Columbia Environmental Research Center, 4200 New Haven Rd., Columbia, MO 65201, USA; 3 Oregon Department of Fish and Wildlife, Oregon Hatchery Research Center, 2457 E. Fall Creek Rd., Alsea, OR 97324, USA

**Keywords:** aquaculture, steelhead, thiamine deficiency, fish health, hatchery

## Abstract

**Simple Summary:**

Steelhead fry reared in hatcheries in Oregon have a high mortality rate while exhibiting some signs of a vitamin B1 (thiamine) deficiency. This study investigates if thiamine supplementation could improve the health and survival of the fry. To do this, adult, female steelhead were injected with thiamine three weeks before spawning; some of the eggs were alternatively treated with a thiamine bath at the time of spawn, and some were treated both ways. The survival and growth efficiency of the thiamine-treated fry were significantly improved compared to fry that were not supplemented with any thiamine. Fry that came from females that were injected with thiamine had greater growth and survival rates than eggs that received thiamine as a bath only; however, any thiamine supplementation improved survival compared to no thiamine supplementation. This is the first description of thiamine deficiency in Oregon’s steelhead.

**Abstract:**

Early rearing of steelhead (*Oncorhynchus mykiss)* in Oregon hatcheries is often problematic; fry can become emaciated and die during the period between hatch and first feed. Thiamine (vitamin B1) deficiency has caused early mortality in salmonids; however, the thiamine status of Oregon’s steelhead populations is unknown, to date. Of the 26 egg samples from three Oregon hatcheries in 2019, 20 (77%) had thiamine levels < 10 nmol/g, and 13 of those samples (50%) had levels <6.5 nmol/g, suggesting the thiamine deficiency of adult, female steelhead. To investigate if thiamine deficiency was causally related to fry survival, females were injected with buffered thiamine HCl 50 mg/kg prior to spawning; additionally, a subset of eggs were supplemented via bath treatment with thiamine mononitrate (1000 ppm) at spawning. Cumulative fry mortality at 8 weeks post-hatch from thiamine-injected females was 2.9% compared to 13.8% mortality of fry without thiamine supplementation. Fry treated only with the thiamine via bath as eggs had a mortality rate of 6.9%. There were no additional improvements for the survival of fry from injected females that also received a thiamine bath. Furthermore, condition factors were greater in thiamine-supplemented fry than in those that received no thiamine. These data identify thiamine deficiency in Oregon steelhead and suggest supplementation with thiamine can mitigate early rearing mortality.

## 1. Introduction

Thiamine, or vitamin B1, is an essential vitamin required in all living organisms for metabolism, growth, immunity, and neurological development and function [1]. Thiamine is an important cofactor necessary for the enzymatic activity of carbohydrate and lipid metabolism [2]. Thiamine deficiency can occur when an animal either does not uptake enough exogenous thiamine, or if the diet contains organisms that produce thiaminase, an enzyme that can be found within tissues of certain fishes and aquatic invertebrates that degrades thiamine [3,4,5]. Thiamine deficiency has been observed across many taxa, including reptiles [6], birds, mussels and mammals [7], and humans [1]. Thiamine deficiency in fishes is a global problem and is responsible for declines in fish populations in the Laurentian Great Lakes [8,9,10], Baltic Sea [11,12], Bulgaria [13], New York Finger Lakes [14], and most recently in California in anadromous Chinook salmon (*Oncorhynchus tshawytscha*) [15]. Thiamine deficiency-related pathologies in fishes are most often observed during the early life stages of development and referred to as the thiamine deficiency complex (TDC) or early mortality syndrome. Adult life stages of fishes can also be affected by TDC, leading to neurological deficiencies, reduced swimming ability, reduced migration, and increased pre-spawn mortality [8,16]. Clinical signs of TDC in juvenile fish include edema, hemorrhage and vascular congestion, hydrocephalus, and enlarged yolk sacs with opacities and residual unabsorbed yolk [17,18]. Histologically, TDC causes the necrosis of brain cells, hepatocellular necrosis with glycogen depletion of liver, and degeneration and glycogen depletion of muscle cells leading to immobilization [19]. More overtly, behavioral aspects of TDC in early life stages are neurological deficiencies observed as an erratic or corkscrew swimming pattern, weakness as observed by an inability to swim up in the water column, underdeveloped vision, and an inability to forage [16,20]. Because yolk thiamine is dependent on the maternal transfer of thiamine into the yolk, the mortality of subsequent fry is often highest (up to 100%) during the first few weeks after hatch while fry rely on their yolk sacs as the primary thiamine source [4].

Thiamine supplementation of fishes can lead to the immediate reversal of clinical signs of TDC, eliminate abnormal behavior, and stop mortality of fry due to TDC [16,17,21]. For these reasons, thiamine supplementation has been used extensively with managed fish populations where TDC has adversely affected fish survival, such as those in the Great Lakes [21]. The most common routes for the administration of thiamine to fish are the injection of gravid females prior to spawning, or the immersion of eggs around the time of fertilization while the shell is still permeable prior to water hardening [16,21,22]. These methods have effectively provided thiamine to the developing embryonic fish to replenish thiamine stores and prevent TDC. 

Steelhead trout (*O. mykiss*) are an important fish species in the Pacific Northwest. They support commercial and tribal treaty fisheries and are an iconic prized fish for recreational anglers. Steelhead express an anadromous life history, spending their first 1–3 years in freshwater, then migrating to the ocean for 1–3 years before returning to freshwater tributaries to spawn. However, unlike most of their anadromous congeners, which invariably die after spawning, some steelhead return to sea after spawning, then return in subsequent years to spawn again (i.e., iteroparity) [23]. In recent years, the Oregon Department of Fish and Wildlife released between 4.5 and 5.5 million steelhead annually from hatcheries into Oregon waters [24]. Hatchery propagation of steelhead augments recreational fisheries and alleviates harvest pressure on wild populations, provides benefits to uphold tribal cultural practices and fishing rights, and contributes to commercial fisheries. At present, 11 steelhead distinct population segments (DPSs) are listed as threatened or endangered under the federal Endangered Species Act (ESA), including four DPSs in Oregon [25].

Rearing steelhead in hatcheries is challenging—fry are easily stressed and hard to start, with a high proportion of “pin-heading” and “drop-out disease” (Reed, pers. observation). These colloquial terms refer to a high mortality rate in the first few weeks after hatching and just after the introduction of feed, with no clear cause of death. This is often diagnosed as a “failure to thrive” and can be associated with 15–30% early rearing mortality (Reed, pers. observation); however, sometimes mortality can be as high as 50%. Affected fry are found on the bottom of rearing tanks near the outflow, weak and emaciated with darkened skin; they may show head swelling or a coagulated yolk remnant in the coelomic cavity. They may also become infected with opportunistic pathogens, such as *Flavobacterium psychrophilum* bacteria, *Saprolegnia* sp. water mold, and *Ichthyobodo sp.* parasites, which contribute to additional fry morbidity and mortality [26].

Despite the elevated mortality of fry with symptoms similar to what has been observed in other species with documented TDC [15,27], potential TDC in steelhead fry from Oregon hatcheries has never been explored. Therefore, our objectives are to investigate the thiamine levels in eggs of returning, mature, female steelhead, and to determine whether thiamine supplementation can affect the mortality or growth of steelhead fry in an Oregon hatchery.

## 2. Materials and Methods

**Egg collection and thiamine supplementation.** In the winter of 2019 (31 January–14 February), samples were collected from three steelhead hatcheries of the Oregon coast: a north-coast facility, Big Creek Hatchery in Astoria (46.1464, −123.5811); a central-coast facility, Alsea Hatchery in Alsea (44.4225, −123.5648); and a south-coast facility, Elk River Hatchery in Port Orford (42.8180, −124.3907). Unfertilized eggs from spawning hatchery steelhead were collected from nine fish at Big Creek Hatchery, ten fish at Alsea Hatchery, and seven fish at Elk River Hatchery. A 10 g egg sample from each female was collected, placed into whirl-pak bags (Nasco Company, Madison, WI, USA), immediately frozen on dry ice, and then maintained at −80 °C until thiamine analysis.

Mature female steelhead were collected and held at Alsea Hatchery in Oregon prior to spawning in January 2022. The estimated average weight of the females in the study was 4 kg and average total length was 62 cm. Each female was placed in either a thiamine treatment group (*n* = 20), which received an injection of thiamine, or a control treatment group (*n* = 14) which received no thiamine. For thiamine injections, thiamine HCl 500 mg/mL (VetOne cat no. 501057, AmerisourceBergen, Boise, ID, USA) was buffered by adding 1.5 mL 10 M NaOH into 10 mL thiamine HCl to achieve a pH of 7.0–7.5. The buffered thiamine solution was used immediately, and any extra buffered thiamine solution was discarded that same day. Adult female steelhead in the treated group were manually restrained, then injected intramuscularly with 1.0 mL buffered thiamine solution (500 mg/female) in the dorsal epaxial musculature using a 23 g needle, tagged with a garment tag in the dorsal fin, and then replaced in the holding pond. Adult female steelhead in the control group were manually restrained, then tagged with a garment tag in the dorsal fin and replaced in their holding pond. All fish were held for 21 days at the hatchery until they were spawned; fish were immobilized via electroshock, and eggs were non-lethally collected via coelomic inflation with manual palpation. A 10 g sample of eggs from 10 control fish and 10 treatment fish was collected into whirl-pak bags and immediately flash-frozen by placement on dry ice, then transferred to a −80 °C freezer for storage until processing for thiamine analysis. The remainder of the eggs from each female were brought to the Oregon Hatchery Research Center in Alsea, Oregon, and fertilized individually in clean 4 L buckets by using milt collected from individual males earlier that morning.

For the immersion treatments, half of the fertilized eggs from each female in the treatment and control groups were treated in an immersion of thiamine mononitrate (PureBulk cat no. 11459, PureBulk, Roseburg, OR, USA) and ambient river water at 1000 ppm for 1 h. Eggs from multiple females in the same treatment groups were pooled and placed into heath trays and rinsed with ambient water for 5 min before being treated with iodophor solution for 30 min, as is the standard protocol for egg-hardening and disinfection. Each group was reared at the Oregon Hatchery Research Center on ambient river water at approximately 19 l/min (5 gal/min) and treated 3 times weekly with formalin at 400 ppm for 15 min to control for external fungal growth. This yielded 4 treatment groups with an estimated 6000–7000 eggs each: eggs from injected females that also received a thiamine bath, eggs from injected females that did not receive a thiamine bath, eggs from non-injected females that received a thiamine bath, and eggs from non-injected females that did not receive a thiamine bath (control group).

**Mortality monitoring and growth measurements.** Daily mortality of fry in each treatment group was monitored, from when the alevins were released (post-hatch) from the heath trays into flow-through fiberglass tanks and recorded for 8 weeks (56 days) as individual deaths per day per treatment group. Additionally, at 8 weeks post-hatch, 75 fish from each group were individually weighed and measured for fork length [28]. The condition factor (K; Equation (1)) was calculated:

(1) $$K=\frac{10^{5}*W}{{FL}^{3}}$$

where *W* is the weight in grams and *FL* is the fork length in mm, which determines the efficiency of the growth and development of fish [29].

**Thiamine Analysis.** Thiamine level in eggs was estimated by a rapid solid phase extraction (SPE) fluorometric method [30] for the 2019 survey of adult steelhead. Briefly, egg samples were preserved by freezing on dry ice and stored in a −80 °C freezer until analysis. Subsamples of 0.5–1.0 g were weighed, homogenized in 2% (*w*/*v*) TCA (trichloroacetic acid), boiled for 10 min, centrifuged at 14,000× gravity for 25 min, and the supernatant applied to a reversed-phase SPE column (Phenomenex, Torrance, CA, USA). The SPE was eluted with methanolic pH 2.05 PO_4_ buffer into two fractions. The thiamine compounds in the two fractions (fraction 1: phosphorylated thiamine vitamers, and fraction 2: non-phosphorylated thiamine) were oxidized to the corresponding thiochromes using alkaline potassium ferricyanide (0.1% K_3_FeCN_6_), and their concentrations were determined fluorometrically (excitation 360 nm, emission 460 nm) on a 96-well plate reader (Biotek Synergy 4, Agilent Technologies, Inc. Santa Clara, CA, USA). Concentrations of phosphorylated (fraction 1) and non-phosphorylated (fraction 2) thiamine were estimated by comparison of the sample fluorescence in each fraction to that of a series of thiamine standards (phosphorylated and non-phosphorylated) taken through the same extraction procedure. Total thiamine was estimated by adding the phosphorylated and non-phosphorylated concentrations of the thiamine vitamers from each sample, normalized to the initial sample weight extracted and reported as nmol/g-egg.

For the 2022 egg analysis, thiamine determination in eggs was conducted by the HPLC method previously described [21,22], with methanol instead of N,N-dimethylformamide (DMF) as the mobile phase. Eggs were preserved and extracted as described above for the SPE method. Following the extraction, thiamine levels were determined using a high-performance liquid chromatograph (HPLC) system (Agilent Technologies 1100 series; Agilent Technologies, Inc. Santa Clara, CA, USA). The HPLC included a delivery pump, automatic sample injector, RP-HPLC column (Agilent Technologies, Inc. Santa Clara, CA, USA) with attached guard column (25 × 2.3 mm; 12 to 20 mm mesh size), and a fluorometric detector (375 nm excitation wavelength and 442 nm emission wavelength for thiochrome detection). The column thermostat was set to 30 °C, and sample injection volume was 80 µL per injection with a total run time of 25 min per sample at a flow rate of 0.6 mL/min. The mobile phase comprised of 6.25 mM potassium phosphate buffer (pH 8.4) with 1% methanol (solvent A) and 100% methanol (solvent B) [31]. A seven-point standard curve with known concentrations of thiamine was generated at the start of each group of samples and interspersed throughout the run.

**Statistical analysis.** All statistics and plots were generated in R v4.1.2 [32]. To compare the thiamine concentrations of steelhead eggs from hatcheries, control vs. injected egg concentrations, we used the permutation test function ‘oneway_test’ in the *coin* package [33] to fit asymptotic K-sample Fisher–Pitman permutation tests. This function is analogous to a one-way ANOVA but permuted 10,000 times so that the *p*-value is the proportion of tests with a value at least as extreme as the ‘true’ test. Permutation tests are useful with small sample sizes because they are insensitive to non-normality and heteroscedasticity. As a post hoc pairwise test across groups, we used the ‘pairwisePermutationTest’ function in the *rcompanion* package [34].

To explore how mortality differed among treatments, we fit non-linear curves to the data to estimate the initial mortality, rate of mortality, and maximum mortality (Equation (2)) using the ‘nls’ and ‘SSasymp’ self-starting asymptotic regression model functions in the *stats* package of base R:

(2) $$f\left(t\right)=y_{f}+\left(y_{0}-y_{f}\right)*e^{-e^{\ln\left(r\right)*t}}$$

where *y_f_* = maximum mortality estimated as the asymptote, *y*_0_ = estimated mortality at day 0 (should be close to 0), *r* = mortality rate or steepness of the curve, and *t* = time since hatch in days.

We compared fish condition (K) using linear models and ‘aov’ in the *stats* package in base R. A test for outliers using the ‘outlierTest’ function in the *car* package [35] indicated 2 of the 300 points in the model may be outliers. We refit the model eliminating these observations, and then tested for differences among groups with Tukey HSD tests using the ‘TukeyHSD’ function in the *agricolae* package [36].

## 3. Results

**Figure 1:**
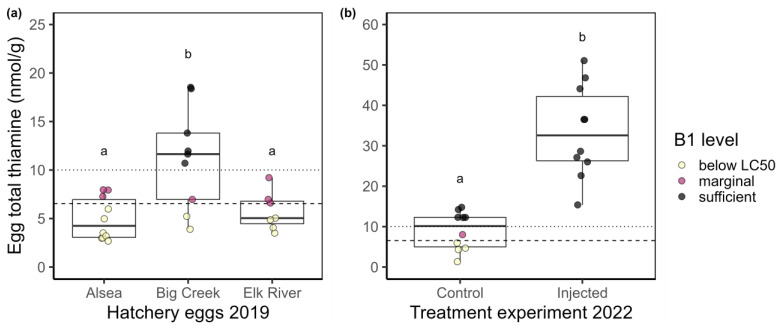
Boxplots (25%, 50%, and 75% quantiles comprise the box) with overlaid raw data points of steelhead egg total thiamine measurements from (**a**) three coastal Oregon hatcheries in 2019, and (**b**) control and injected female eggs from 2022. Egg concentrations are colored based on thiamine concentration thresholds. Yellow points are below LC50 for steelhead of 6.54 nmol/g (dashed line [37]), red points lie between LC50 and the value considered sufficient (10 nmol/g, dotted line [38]), and black points represent egg thiamine concentrations that are likely sufficient for early growth and development as determined from individual-based models for recruitment to lake trout populations [38]. Different letters (a or b) indicate post hoc permutation tests suggest group differences at *p* < 0.05. Note the y-axes have different scales.

**2019 egg thiamine.** Eggs from the northern-coast (Big Creek) hatchery had total thiamine values that ranged from 3.9 to 18.5 nmol/g with a mean of 11.2 nmol/g (*n* = 9). Eggs from the central-coast (Alsea) hatchery had total thiamine values that ranged from 2.7 to 9.8 nmol/g with a mean of 4.9 nmol/g (*n* = 10). Eggs from the south-coast (Elk River) hatchery had total thiamine values that ranged from 3.5 to 9.2 nmol/g with a mean of 5.8 nmol/g (*n* = 7; Figure 1a). Permutation tests indicated that there were differences in egg thiamine among hatcheries (chi-squared = 10.623, d.f. = 2, *p*-value = 0.005). Pairwise tests indicated the egg samples from fish at the northern-most facility, Big Creek Hatchery, had statistically higher total egg thiamine than eggs from the other two hatcheries (Figure 1a).

**2022 egg thiamine.** Female, adult steelhead tolerated the injections well with no mortality and no apparent adverse effects from the thiamine injections. Eggs from the control group had an average total thiamine level of 9.0 nmol/g (range 1.3–14.8), whereas eggs from the injected group had an average total thiamine level of 33.5 nmol/g (range 15.4–51.1; Figure 1b). The statistical difference between these groups (Z = −3.60, *p*-value = 0.0003) strongly suggests that the injection of thiamine effectively transferred thiamine into the egg after 21 days.

**Figure 2:**
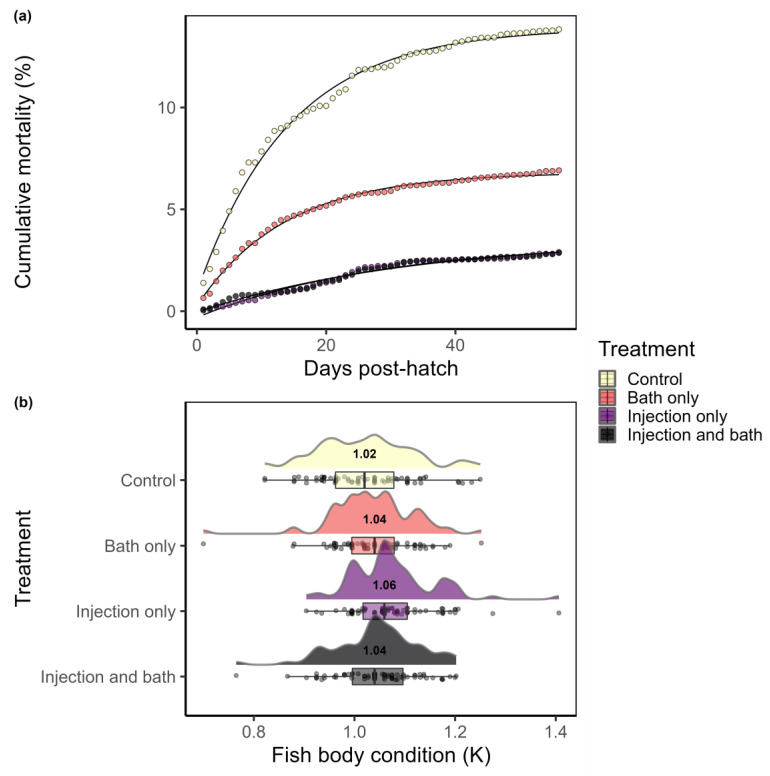
(**a**) Cumulative mortality was assessed daily (points) for 56 days post-hatch in the four treatments with model included as a line; endpoints are control 13.8%, bath only 6.9%, and injection only and injection and bath 2.9%; (**b**) fish body condition, K, of fry 8 weeks post-hatch (*n* = 75 each treatment). All data are included overlaid as points on the horizontal boxplot to show the distribution of the data (25%, 50%, and 75% quantiles are included in the box). Overlaid on top of each boxplot is a probability density function to show the density of points at each condition factor with the median value expressed under each curve.

**Mortality.** The highest survival rate was observed in the groups that came from injected females; fry from thiamine-injected females or fry from thiamine-injected females that also received thiamine as a bath immersion at fertilization had an overall cumulative mortality 4× lower than the control group. Fry in the control group had a cumulative mortality rate of 13.8% during the first 8 weeks after hatch (Figure 2a, Table 1). Fry from non-injected females that received thiamine as a bath treatment at fertilization had a cumulative mortality rate of 6.9%. Fry from injected females and fry from injected females that also received thiamine as a bath had similar cumulative mortality rate of 2.9% (Figure 2a; Table 1).

The non-linear mortality model (Equation (2)) showed that fry that had received thiamine supplementation had lower early life stage mortality than control fish (Table 1). Estimated and measured cumulative mortality rates were very similar (Figure 2a, Table 1). Fry groups from fish that received injections had half the mortality rate parameter estimate (rate ≈ 0.03) than fish that did not receive any thiamine supplementation or had a bath treatment only (rate ≈ 0.07; Table 1). Overall, thiamine supplementation improved mortality in steelhead fry, and an injection of thiamine to the gravid females had a greater positive effect on survival than a bath immersion treatment of eggs at fertilization.

**Condition**. ANOVA tests indicated there were no statistical differences between treatment groups for average body mass (F-statistic_3, 296_ = 0.92, *p* = 0.43) or average fork length (F_3, 296_ = 0.50, *p* = 0.68; data available [39]). However, fry from thiamine-injected females had a higher mean condition factor (K) compared to the other groups (F_3, 296_ = 5.49, *p* = 0.001; Figure 2b). Average condition factor (K) of fry from the injection-only group was 1.07. Post hoc tests suggested that the injection-only fry had significantly higher body condition (K) rates than the fish from the control (mean K = 1.02, *p* = 0.001) and bath-only (mean K = 1.04, *p* = 0.041) groups, but were statistically equivalent to the group from injected females that also received a bath (mean K = 1.05, *p* = 0.164; Figure 2b). These data suggest that at 75 days post-hatch, fry from thiamine-injected females were growing at a more efficient rate than fry that did not receive thiamine.

## 4. Discussion

These data revealed that thiamine concentrations in steelhead eggs from three Oregon hatcheries were frequently below levels associated with thiamine-related mortality in this species. When the egg thiamine LC50 for steelhead proposed by Futia and Rinchard [37] was applied (dashed line in Figure 1), 50% (13/26) of the eggs sampled had total thiamine concentrations below the LC50 of 6.54 nmol/g. Sublethal effects of TDC have been observed in lake trout when egg thiamine levels were as high as 10 nmol/g [38]; extrapolating this value of 10 nmol/g (dotted line in Figure 1) to these results suggests that 70% of the eggs sampled could experience mortality or sublethal effects associated with TDC. Although the egg thiamine levels were higher in the northern-most hatchery as compared to the central and southern hatcheries (Figure 1a), the surveillance of egg thiamine levels at additional locations should be investigated to further elucidate geographic patterns of egg thiamine levels in steelhead populations.

The improved condition factor for thiamine-supplemented fry found in this study further suggests thiamine deficiency affects Oregon hatchery steelhead performance. Fitzsimons and colleagues [40] found low egg thiamine concentrations resulted in decreased fry growth rates, reduced predator avoidance, increased vulnerability to prey, and consequently reduced feeding and foraging behavior. The results of this study are consistent with previous work suggesting growth can be the most evident and a sensitive metric in developing salmonid fry experiencing thiamine deficiency [4,40]. The differences in condition factors for thiamine-supplemented compared to thiamine-deficient fish would undoubtedly become more apparent as the fish continue to grow.

The dominant paradigm for causes of TDC is that high amounts of thiaminase-containing prey in the diets causes the depletion of thiamine in predatory fishes [5,41]; yet, the etiology of thiamine deficiency in Oregon’s steelhead populations is unclear. In early 2020, Chinook salmon off the coast of California suffered elevated mortality caused by TDC [15], which was proposed to be caused by the availability of prey and increased ingestion of thiaminases from clupeids, such as Northern anchovy (*Engraulis mordax*). Oregon steelhead, however, express different life histories and ocean migration patterns than Chinook salmon; there is a considerable variation in the migration patterns and timing of Oregon Coastal and Lower Columbia River steelhead populations, and they have a rapid off-shore migration into open oceans as juvenile fish [42]. Steelhead are opportunistic and selective predators with a varied diet that consists of cephalopods, fish, and invertebrates; diets have been reported to consist of less than 5% clupeids during their marine life stage with adjustments to diet observed during warm ocean years [43,44]. However, juvenile fish and kelts (that is, fish that have already spawned) have been shown to consume high proportions of anchovy in their diet while offshore [45]. The roles of thiamine, thiaminases, and the marine environment are poorly understood as they relate to the life history of steelhead trout and are a rich area for future investigations.

Low thiamine in steelhead is an additional stressor on already struggling populations. A major factor that affects fish survival in hatcheries is infectious disease [26,46]. Thiamine deficiency contributes to T-cell-dependent and -independent immune dysfunction [47], as well as lowered WBC bactericidal activity and the mitogenesis of fish cells in vitro [48]. Whether the thiamine supplementation of steelhead adults and fry affects their immune status and ability to combat infectious disease agents in the hatchery remains unresolved. Another major threat to steelhead populations in Oregon, and elsewhere, is habitat degradation and loss [49,50]. Climate change is already influencing the success of steelhead in Oregon, as steelhead have been shown to be highly sensitive to the risks of climate change [50]. Changing ocean temperatures and prey availability will continue to affect steelhead ocean distributions [49], but also likely influence disease dynamics and thiamine status [15]. Whether these multiple stressors are additive or synergistic is an important consideration in evaluating the long-term viability of steelhead in Oregon. If the hatchery steelhead sampled in this study are representative of wild steelhead populations in Oregon, thiamine deficiency may be an important and underappreciated limiting factor for their survival.

## 5. Conclusions

These results show steelhead returning to the coastal hatcheries in Oregon have low thiamine levels that affects the survival and body condition of their progeny. Thiamine levels were higher in eggs from females returning to the northern-most hatchery sampled. Supplementing progeny with thiamine improved survival rates during the first few weeks of life. Thiamine injection of adults prior to spawning had the greatest benefit to the survival and body condition of offspring; however, egg thiamine supplementation in a bath also increased survival. Providing supplemental thiamine can profoundly increase the survival, growth, and health of steelhead fry in Oregon hatcheries. This is the first description of the thiamine deficiency of steelhead populations in Oregon.

## Figures and Tables

**Table 1:** Model parameters from function to estimate mortality (%) within each treatment. *y*_0_ is the initial mortality on day 0; all treatments were very close to zero initial mortality. Ln(rate) and rate describe the steepness of the mortality curve in our models, expressed both as the model uses it (natural log of rate) and back-transformed to simply a rate for ease of interpretation (rate); *y_f_* is the asymptote of cumulative mortality. Each model was fit to the daily mortality from that treatment (*n* = 56, model df = 53), and total residual standard error of the model is in the last column.

Parameter	Estimate	SE	t-Value	*p*-Value	Residual Standard Error
**Control**					0.0031
*y_f_*	13.907	0.105	132.59	<0.00001	
*y*_0_	0.935	0.198	4.72	0.00002	
ln(rate)	−2.653	0.034	−77.73	<0.00001	
rate	0.070				
**Bath only**					0.0011
*y_f_*	6.814	0.036	188.87	<0.00001	
*y*_0_	0.269	0.072	3.76	0.00042	
ln(rate)	−2.622	0.024	−109.71	<0.00001	
rate	0.073				
**Injection only**					0.0013
*y_f_*	3.434	0.140	24.49	<0.00001	
*y*_0_	−0.305	0.070	−4.36	0.00006	
ln(rate)	−3.360	0.088	−38.01	<0.00001	
rate	0.035				
**Injection and bath**					0.0013
*y_f_*	3.604	0.190	18.93	<0.00001	
*y*_0_	−0.046	0.068	−0.68	0.502	
ln(rate)	−3.528	0.110	−32.16	<0.00001	
rate	0.029				

## Data Availability

The data are available via U.S. Geological Survey data release [39] https://doi.org/10.5066/P9KDEUBK, accessed on 1 December 2022.
